# Brain functional connectivity under teleoperation latency: a fNIRS study

**DOI:** 10.3389/fnins.2024.1416719

**Published:** 2024-11-13

**Authors:** Yang Ye, Tianyu Zhou, Qi Zhu, William Vann, Jing Du

**Affiliations:** ^1^ICIC Lab, Engineering School of Sustainable Infrastructure & Environment, University of Florida, Gainesville, FL, United States; ^2^National Institute of Standards and Technology, Boulder, CO, United States

**Keywords:** human-robot interaction, sensory manipulation, haptic feedback, latency mitigation, motor functions, functional near-infrared spectroscopy (fNIRS)

## Abstract

**Introduction:**

Long-distance robot teleoperation faces high latencies that pose cognitive challenges to human operators. Latency between command, execution, and feedback in teleoperation can impair performance and affect operators’ mental state. The neural underpinnings of these effects are not well understood.

**Methods:**

This study aims to understand the cognitive impact of latency in teleoperation and the related mitigation methods, using functional Near-Infrared Spectroscopy (fNIRS) to analyze functional connectivity. A human subject experiment (*n* = 41) of a simulated remote robot manipulation task was performed. Three conditions were tested: no latency, with visual and haptic latency, with visual latency and no haptic latency. fNIRS and performance data were recorded and analyzed.

**Results:**

The presence of latency in teleoperation significantly increased functional connectivity within and between prefrontal and motor cortexes. Maintaining visual latency while providing real-time haptic feedback reduced the average functional connectivity in all cortical networks and showed a significantly different connectivity ratio within prefrontal and motor cortical networks. The performance results showed the worst performance in the all-delayed condition and best performance in no latency condition, which echoes the neural activity patterns.

**Conclusion:**

The study provides neurological evidence that latency in teleoperation increases cognitive load, anxiety, and challenges in motion planning and control. Real-time haptic feedback, however, positively influences neural pathways related to cognition, decision-making, and sensorimotor processes. This research can inform the design of ergonomic teleoperation systems that mitigate the effects of latency.

## 1 Introduction

Robotic teleoperation, which involves a human operator controlling an intelligent system from a distance, has gained increasing popularity in difficult, dangerous, and less accessible tasks (Neumeier et al., 2019). One of the critical needs in this area is to advance understanding of how latency affects human operators and the effectiveness of related mitigation methods. Latency in robot teleoperation is a prevalent, often inevitable issue and poses significant challenges to operators and system designers (Neumeier et al., 2019). Robot teleoperation latency of more than 50 ms can significantly increase the occurrence of overshoot and oscillations (Kapoor et al., 2003; Richter et al., 2019). While roundtrip cross-continental communication between London and Toronto can lead to about 560-ms latencies (Nguan et al., 2008) and solely the signal transmission time in moon operations takes around 2,560 ms (NASA, 2023), latency is a significant concern in long-distance robot teleoperation (Larcher et al., 2023), which may result in reduced performance, decreased user satisfaction, and even safety issues (Neumeier et al., 2019). Latency can stem from various sources, including hardware limitations and the physical speed of signal transmission.

Mitigation approaches have been proposed to counteract the impact of latency when hardware and transmission technologies reach their limits, such as the predictive approach (Brudnak, 2016), adaptive control (Shahdi and Sirouspour, 2009), and multi-sensory feedback (Su et al., 2023). Predictive control strategies involve anticipating the system’s future state to compensate for the delay (Brudnak, 2016; Sirouspour and Shahdi, 2006). Adaptive control techniques aim to adapt the robot’s behavior in real-time to accommodate latency (Shahdi and Sirouspour, 2009), and multi-sensory feedback combines multiple sensory modalities to enhance the operator’s perception and control capabilities (Su et al., 2023). Among all approaches for mitigating the behavioral and cognitive consequences of teleoperation delays, the multi-sensory integration method stands out as a promising solution. Unlike other methods that rely on automation and adaptive algorithms, multi-sensory control leverages sensory information to enhance the operator’s subjective experience (Triantafyllidis et al., 2020; Toet et al., 2020), thus facilitating human adaptation to time-delayed teleoperation. Although these findings provide promising evidence for identifying a novel mitigation method for improving the performance in delayed teleoperation, it is less clear how these multi-sensory variations impact the operator’s neural functions as a possible cause of changed motor and cognitive performance. Especially, the neural basis of how operators integrate synthetic haptic feedback with other sensory modalities at the neurofunctional level remains underexplored. Understanding the neural mechanisms that underlie this integration is crucial for designing more effective user interfaces. This would not only enhance immediate task performance but also ensure sustainable cognitive adaptation in other teleoperation scenarios. There is a need for exploring the changes in neural activity and cognitive processing associated with multi-sensory feedback in teleoperation tasks. The following section provides details regarding the state-of-the-art methods and theories for latency mitigation in long-distance robot teleoperation.

## 2 State-of-the-art

Researchers investigated multiple methods to mitigate latency when hardware reaches the limit. Classic latency mitigation approaches utilize mathematical modeling and control algorithms to smoothen actions to mitigate the overshoot and oscillations due to delay (Farajiparvar et al., 2020). For example, Sirouspour and Shahdi (2006) proposed a multi-model predictive controller using a discrete linear quadratic Gaussian (LQG) approach for teleoperation with time delay. To reduce computational load and avoid numerical issues, the sampling rate was lowered as delays increased, but this method risks compromising closed-loop response and teleoperation stability. Polat and Scherer (2012) formulated an integral quadratic constraints framework to analyze the stability of uncertain bilateral teleoperation systems with network theory, which is further extended by Tugal et al. (2016) to time-invariant and time-variant latencies. Another category of latency mitigation focuses on time-series prediction using statistical models and neural networks. For example, Aburime et al. (2019) identify the uncertain latencies and predict target waypoints based on recursive least squares filtering. Chen et al. (2020) utilizes multivariate linear regression to forecast delay in space. Similar research also shows the great potential of other methods in time-series prediction, such as auto-regressive models (Hu et al., 2012), recurrent neural networks (Kombrink et al., 2011), and convolutional short-term memory (Karim et al., 2017). These methods predict the occurrence of latency and the delayed actions, with the assumption that the delayed actions follow a similar pattern of previous actions, which may not be effective in unstructured environments. Other than the control side of robot teleoperation, another promising approach of latency mitigation focuses on the human side using multi-sensory integration. Richter et al. (2019) leveraged augmented reality to render the predicted status of remote surgical robots to human operators and found a significant increase in task completion time while maintaining the same error rate. Tsokalo et al. (2019) further propose a digital twins approach that mirrors remote operation and provides low latency visual feedback from digital twins prior to receiving the real visual data. This human-in-the-loop approach demonstrates feasible solutions for critical robot teleoperations. Du et al. (2023) further extended the concept through the integration of modified haptic feedback, which plays a crucial role in mitigating the challenges associated with latency, such as robustness, reliability, and trust issues. In one of our previous studies (Du et al., 2023). Du et al. (2023) designed and tested a system that generated synthetic, simulated haptic feedback, coupled, or decoupled with the visual feedback in a teleoperation task for NASA’s R&R (replacement and repair) task. They found that providing synthetic haptic feedback (e.g., the momentum of moving an object in the direction of acceleration) immediately after the motor actions in real time could bring a variety of performance and perceptual benefits, such as increased accuracy of object placement, reduced time on tasks, and shorter perceived delays (Du et al., 2023).

While these methods were reported effective for mitigating the impact of teleoperation latency from the human operator side, the cognitive impact of these mitigation methods remains unclear. These methods alter the human operator’s perception via simulated visual or haptic cues, which raises concerns regarding cognitive status. These concerns primarily stem from how the brain interprets these sensations, potentially leading to cognitive dissonance, sensory overload, or perceptual maladaptation (Eckstein et al., 2020). For example, altered sensations can also cause over-trust in the system, where users rely too heavily on artificial feedback (Lewis et al., 2018; Ullrich et al., 2021). This is particularly dangerous in high-stakes environments like teleoperation in surgery or industrial machinery. In addition, the altered sensation may conflict with other sensation channels, for example, the misaligned visual and haptic feedback (Du et al., 2023), asynchronized result feedback, and visual feedback (Tsokalo et al., 2019). Existing psychological and neurological studies suggest that the altered or misaligned feedback may disrupt the brain’s predictive coding process (Eckstein et al., 2020) and mismatch negativity response (Xu et al., 2019), leading to confusion, sensory-motor mismatches, or incorrect responses. As such, understanding the cognitive and neurological impact of altered sensory feedback is particularly important.

This study aims to bridge the research gap between the increasing occurrence of latency in teleoperation and the limited understanding of its cognitive impact and related mitigation methods. To achieve this, we conducted a simulated robot arm pick-and-place task within a physics engine, introducing data transmission latency. Participants were tasked with operating the robot through a haptic device while observing camera views from a control deck. Three controlled conditions were tested: a no-latency condition (NL), a delayed condition with synchronized latency (Syn), and an anchoring condition (Anc) (Du et al., 2023). NL condition simulated an optimal condition that transmitted the haptic and visual feedback with no additional latency. Syn condition represented a synchronized data transmission strategy in which visual and haptic feedback were transmitted in the same batch with the same latency. Anc simulated an asynchronized data transmission with a simulation strategy (Du et al., 2023), which simulates low-rank haptic feedback in local workstations, thus providing real-time haptic feedback. Anc condition was essentially a latency mitigation strategy in which we manipulated the users’ sensory feelings to create a subjective feeling of less delay.

To investigate the cognitive impact of latency and the effectiveness of mitigation methods, this study utilizes Functional Near-Infrared Spectroscopy (fNIRS) for analyzing brain activities and underlying cognitive behaviors (Tak and Ye, 2014). Compared to medical neuroimaging techniques relying on neurovascular coupling, such as functional magnetic resonance imaging (fMRI) (Ochsner et al., 2002; Logothetis, 2008) and positron emission tomography (PET) (Andreasen et al., 1996), fNIRS demonstrates better portability and flexibility in field research. In addition, unlike sensors based on the electromagnetic activity of the brain, such as electroencephalography (EEG) (Ray and Cole, 1985) and magnetoencephalography (or MEG) (Halgren et al., 2000), fNIRS is more robust for motor artifacts (Vitorio et al., 2017) because it tracks cortical hemodynamics that is less sensitive to body movements (Pinti et al., 2018). These features of fNIRS make it suitable for monitoring the operator’s neural activities. fNIRS measures hemodynamic responses, including oxy-hemoglobin (HbO), deoxy-hemoglobin (Hb), and total hemoglobin (THb), providing insights into neural processing. Previous studies have demonstrated the utility of fNIRS in brain functional connectivity research (Nguyen et al., 2018; Tyagi et al., 2023), revealing networks within the sensorimotor, visual, auditory, and language systems during various tasks. Analyzing brain connectivity through fNIRS offers reliable and valuable insights into cognitive processes. A human-subject experiment involving 41 participants was conducted to collect performance data and fNIRS measurements. Functional connectivity analysis was performed to gain insights into neural activities within and between brain regions in response to latency and mitigation strategies. This research aims to provide valuable contributions to the field of teleoperation by shedding light on the cognitive aspects of latency and offering insights into effective mitigation techniques.

## 3 Methods

### 3.1 Participants

We recruited 41 healthy subjects (college students) to participate in this experiment. Among all participants, there were 26 males (63.41%) and 15 females (36.59%). The average age was 25.12 (*σ* = 9.34). A total of 18 were with engineering backgrounds and majored in civil engineering or mechanical engineering, while others were self-identified as non-engineering students. In addition, 12 participants (29.27%) self-identified with previous experience with VR, while others claimed a lack of experience with VR. To be noted, our post-experiment analysis did not find any difference among these demographic or experience groups. This research complied with the American Psychological Association Code of Ethics and was approved by the Institutional Review Board at University of Florida. Informed consent was obtained from each participant.

### 3.2 Experiment environment

**Figure 1:**
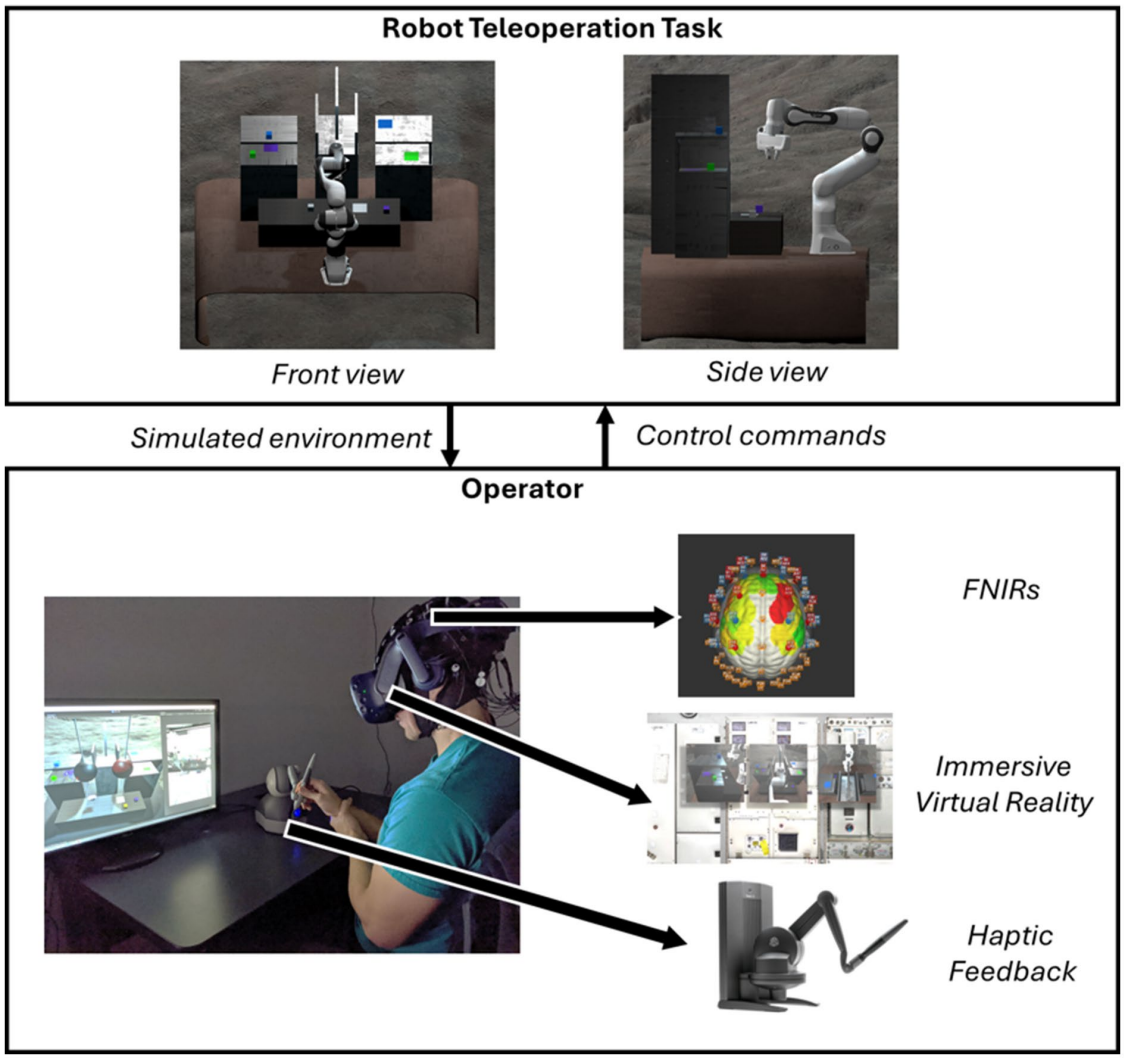
The experiment environment for this research.

To perform a controlled experiment, we built the experiment task in virtual reality (VR) in the Unity game engine (Zhou et al., 2023a), as shown in Figure 1. The simulated environment is robot teleoperation on the moon, in which a human operator controls a remote robot via a teleoperation station with monitors and controllers. VR provides an immersive simulated environment. Three simulated monitors were placed on the wall, providing front, side, and top-down views. Participants sat in front of the screens and manipulated a haptic controller, TouchX (3DSystems, 2022). The position and orientation of TouchX’s end effector were mapped to the remote robot’s end effector. Meanwhile, the pressure that participants exerted on TouchX’s stylus was mapped to the remote robot’s gripping force. The study was performed in a quiet room with constant light conditions. With VR and fNIRS sensors on, participants sat stably on a chair in front of a table and steadily grabbed TouchX’s stylus. The design details of hardware and software systems, including the coupling of VR and haptic devices and robot arms, robot contact simulation, and inverse kinematics calculation, can be found in our previous papers (Zhou et al., 2023a; Ye et al., 2023a; Zhou et al., 2023b; Ye et al., 2023b). In short, game engine simulates the virtual environment and physical interactions, including gravity and collision. The simulated physical interactions inform TouchX of generating haptic feedback that corresponds to the anticipated physical interactions occurring in the teleoperated environment. The virtual robot’s motion is calculated using Final IK (RootMotion, 2023) for realistic robotic gesture control.

### 3.3 Task design

**Figure 2:**
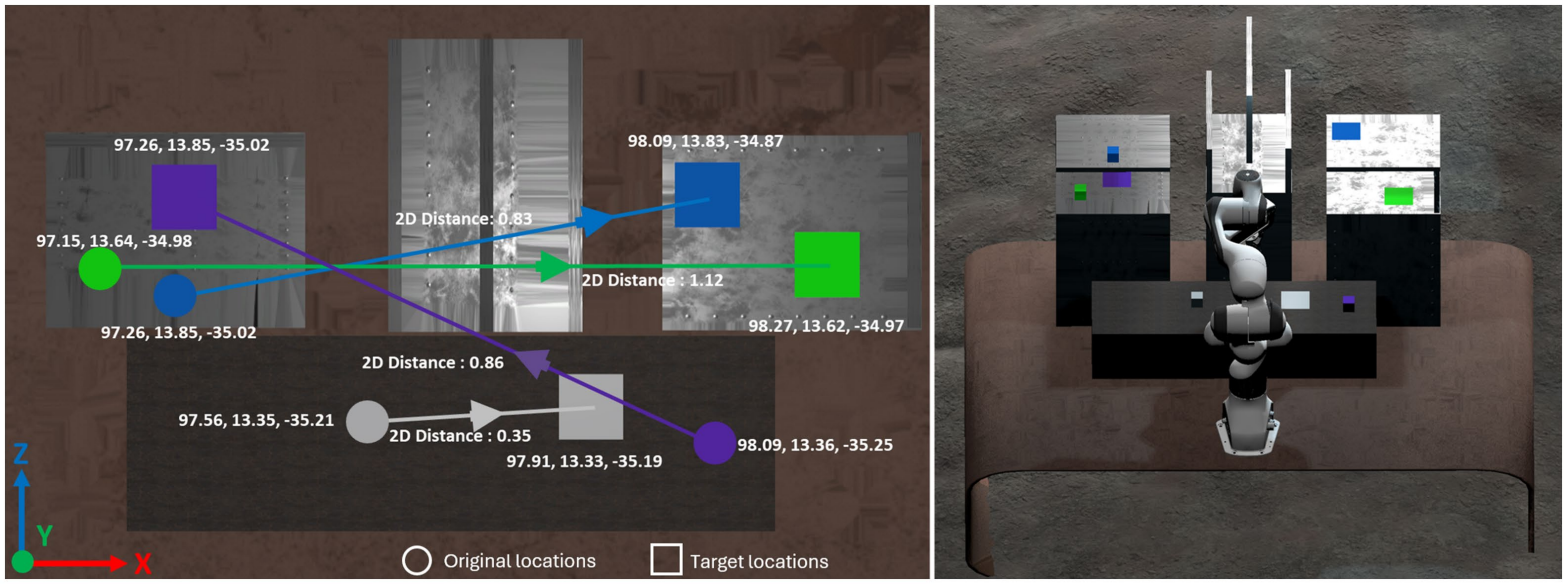
The layout of the object manipulation task in the experiment.

The objective of the teleoperation task was to grab and place objects in the correct position. There were four cubes with distinctive colors. The cubes were placed at fixed original locations (indicated by the circular marks in Figure 2) and should be moved to the corresponding target locations (indicated by the square marks in Figure 2). The target locations were highlighted in VR with the same color of the corresponding cubes. The purpose was to show clearly where each cube should be moved. Participants had to pick up the cubes and place them on the corresponding targets in a fixed order for a controlled experiment. Figure 2 illustrates the layout of the experiment task. The intra-cube distance and varied between different cube-target pairs, with obstacles strategically placed along the movement trajectories. These obstacles, differing in size and position, added complexity to the task, representing varying movement challenges that participants had to navigate. The task setup is invariant to different participants for a controlled experiment.

### 3.4 Latency design and experiment conditions

For a single discrete event, there are three latencies in this experiment. First, the latency between an action initiation by the participant and the execution by the robot ($\Delta_{o}$). This latency accounts for the command transmission lag from participants to the remote robot, and the time required for running inverse kinematics to calculate variable robot joint parameters. Second, the latency between the robot’s haptic status ($\Delta_{h}$), such as touching an obstacle, and the haptic feedback received by participants. Similarly, there is a latency between capturing the visual information around the robot and the participant receiving the visual information in the workplace ($\Delta_{v}$). A time-series combination of these time points forms the event design with latency (S), as described in Equation 1.


(1) $$S={\left(t_{a},t_{r},t_{h},t_{v}\right)}^{T}={\left(t_{a},t_{a}+\Delta_{o},t_{a}+\Delta_{o}+\Delta_{h},t_{a}+\Delta_{o}+\Delta_{v}\right)}^{T}$$

where $t_{a},t_{r},t_{h},t_{v}$ are the continuous time in which actions is initiated, executed, triggered haptic feedback, and triggered visual feedback. Different combinations of latency values form different conditions in this experiment. We take relevant conditions from the experiment, specifically:

NL condition assumes neglectable transmission and processing latency ($\Delta_{o}\to 0$ and $\Delta_{h}=\Delta_{v}=\Delta_{o}$). In this condition, haptic and visual feedback both happen immediately after an action initiated by the human operator, i.e., in real-time. Note that although this condition is named as No-Latency for simplicity, there is latency due to system processing ($\Delta_{o}$ ≠ 0) in the experiment. However, the latency is neglectable to human operators because VR update frequency is 90 Hz, which corresponds to a neglectable 11 ms intervals. This simulates an optimal teleoperation scenario without the impact of significant latency. The latency design matrix is defined in Equation 2.


(2) $$S_{NL}=\lim_{\Delta_{o}\to 0}{\left(t_{a},t_{a}+\Delta_{o},t_{a}+2\Delta_{o},t_{a}+2\Delta_{o}\right)}^{T}$$

Anc condition assumes no delays for haptic feedback, and non-neglectable latency for visual feedback. This condition transmits haptic stimulation as soon as possible via simulated synthetic haptic cues. Due to data size, transmitting and decoding visual feedback takes longer. In this experiment, we take $\Delta_{v}$ ranges from 100 to 850 ms. Specifically, three trials are included in Anc condition, with $\Delta_{v}$ = 100, 475, and 850 ms, respectively. The latency within a trial remains constant. We intend to mitigate the subjective feeling of latency through no-latency haptic feedback. This condition can be defined in Equation 3.


(3) $$S_{Anc}=\lim_{\Delta_{o}\to 0}{\left(t_{a},t_{a}+\Delta_{o},t_{a}+\Delta_{o},t_{a}+\Delta_{o}+\Delta_{v}\right)}^{T}$$

Syn condition assumes non-neglectable latency and the same for both visual and haptic feedback. In this experiment, we take $\Delta_{o}=150$ ms, $\Delta_{v}=\Delta_{h}\in \left[100\;ms,850\;\mathrm{ms}\right]$. Specifically, three trials are included in Anc condition, with $\Delta_{v}=\Delta_{h}$= 100, 475, and 850 ms, respectively. The values of latency are based on the Gateway to moon communication time and accounts for a range of processing time (Kumar et al., 2020). The feedback latency is consistent in each trial. In this condition, the haptic feedback is intentionally delayed, matching the delayed visual feedback to create synchronized feedback. In other words, what the users feel (through haptic feedback) is synchronized with what the users see (through visual feedback) in Syn condition. The rationale is to ensure multisensory congruency, i.e., a coherent representation of sensory modalities to enable meaningful perceptual experiences. This condition can be expressed as Equation 4.


(4) $$S_{Syn}={\left(t_{a},t_{a}+\Delta_{o},t_{a}+\Delta_{o}+\Delta_{v},t_{a}+\Delta_{o}+\Delta_{v}\right)}^{T}$$

The experiment was designed as a within-participant experiment, i.e., each participating subject experienced three conditions. To avoid learning effects, the sequence order of conditions and trials within each condition was shuffled for each subject. Participants were told that latency issues existed in the experiment, but they did not know the latency configurations in each trial. The performance data (time and accuracy), motion data (moving trajectory), eye tracking data (gaze focus and pupillary size), and neurofunctional data (measured by fNIRS) were collected. Participating subjects were also requested to report their perceived delays to compare them with actual ones. Before the experiment, each participant was required to fill out a demographic survey and the consent form approved by UF’s IRB office. Then, they would take a 10-min training session to familiarize themselves with VR. Afterward, participants were required to take a break of 5 min by sitting quietly with all sensors on. This break session was for collecting fNIRS baseline data and removing possible impacts of the training session.

### 3.5 Performance data analysis

We first analyzed the performance of the object manipulation, including time on task measured in seconds and placement error measured in cm. Time on task is the difference between the end and start times, measuring the completion speed. The placement error is calculated as the Euclidean distance between the cube’s actual placement and the target location’s center. The smaller this distance, the more accurate the placement. Without further notes, all analyses are based on the aggregated data of four cubes.

Then, we analyzed the accuracy of subjective delay estimation. Two metrics were analyzed: visual perception difference and haptic perception difference. Participating subjects were also requested to report their perceived latency after each trial. While collecting perceived latency, we verbally asked participants the questions “How much is your perceived visual latency?” and “How much is your perceived haptic latency?” The reported numerical values were recorded. Numerical values were preferred to the scale value questionnaire because numerical values in seconds/milliseconds are more direct indicators of perceived latency, while scales are hard to anchor across different participants. We calculated perception difference by subtracting perceived latency from actual latency in the corresponding trials. These evaluation metrics provide a comprehensive evaluation of the objective performance and subjective feeling of latencies.

### 3.6 fNIRS setup and analysis

Functional Near-Infrared Spectroscopy has proved to be an effective and scalable tool that measures the changes in oxygenated and deoxygenated hemoglobin concentration of the brain’s cortical regions (Wilcox and Biondi, 2015) at a similar level to fMRI (Nguyen et al., 2018). Previous studies have demonstrated the significant correlation between functional connectivity measured by fNIRS among cortical areas and the level of depression and anxiety (Zhang et al., 2022), cognitive load (Newton et al., 2011), creativity (Wei et al., 2014), and certain disease severity (Wang et al., 2014). In this study, we are particularly interested in the impact of teleoperation latencies on cognitive and motor coordination functions. Previous studies suggest that the prefrontal cortex is the cognitive center of the brain, and the activity within the region is an indicator of mental workload and levels of anxiety and depression (Zhang et al., 2022). The motor cortex is believed to be the major cortical region facilitating motor control and coordination (Machado et al., 2010; Koch et al., 2006), which is the main contributor to generating neural impulses that pass down to the spinal cord and control movement execution (Rizzolatti et al., 2002). As such, we selected the prefrontal cortex and motor cortex as the central regions of interest.

**Figure 3:**
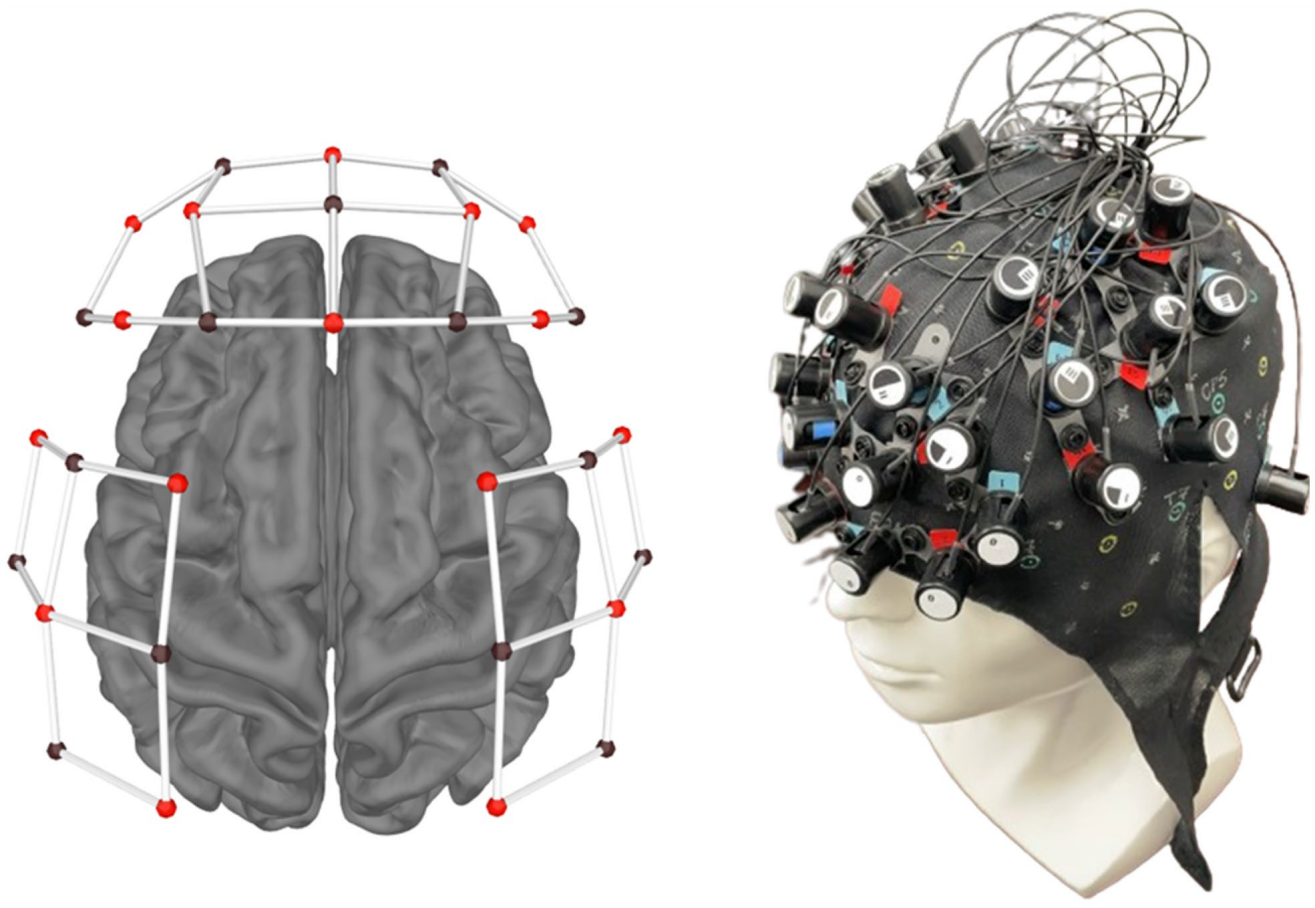
fNIRS setup in this experiment.

The fNIRS head cap (NIRx, 2023) used in this study had 16 sources and 15 infrared light detectors, forming 40 channels. An additional detector was placed at the right pre-auricular point (RPA) as a reference point. The probe map is shown in Figure 3, visualized by MNE. The fNIRS setup covers the prefrontal cortex and motor cortex. Specifically, fNIRS in this study covered seven regions within the prefrontal and motor cortex defined in Brodmann areas: anterior prefrontal cortex (APFC), left dorsolateral prefrontal cortex (LDLPFC), right dorsolateral prefrontal cortex (RDLPFC), left premotor cortex (LPM), right premotor cortex (RPM), left primary motor cortex (LM1), and right primary motor cortex (RM1). APFC, LPFC, and RPFC were merged to evaluate prefrontal cortical activities. LM1, RM1, LPM, and RPM were merged to assess the motor cortex activities.

**Figure 4:**
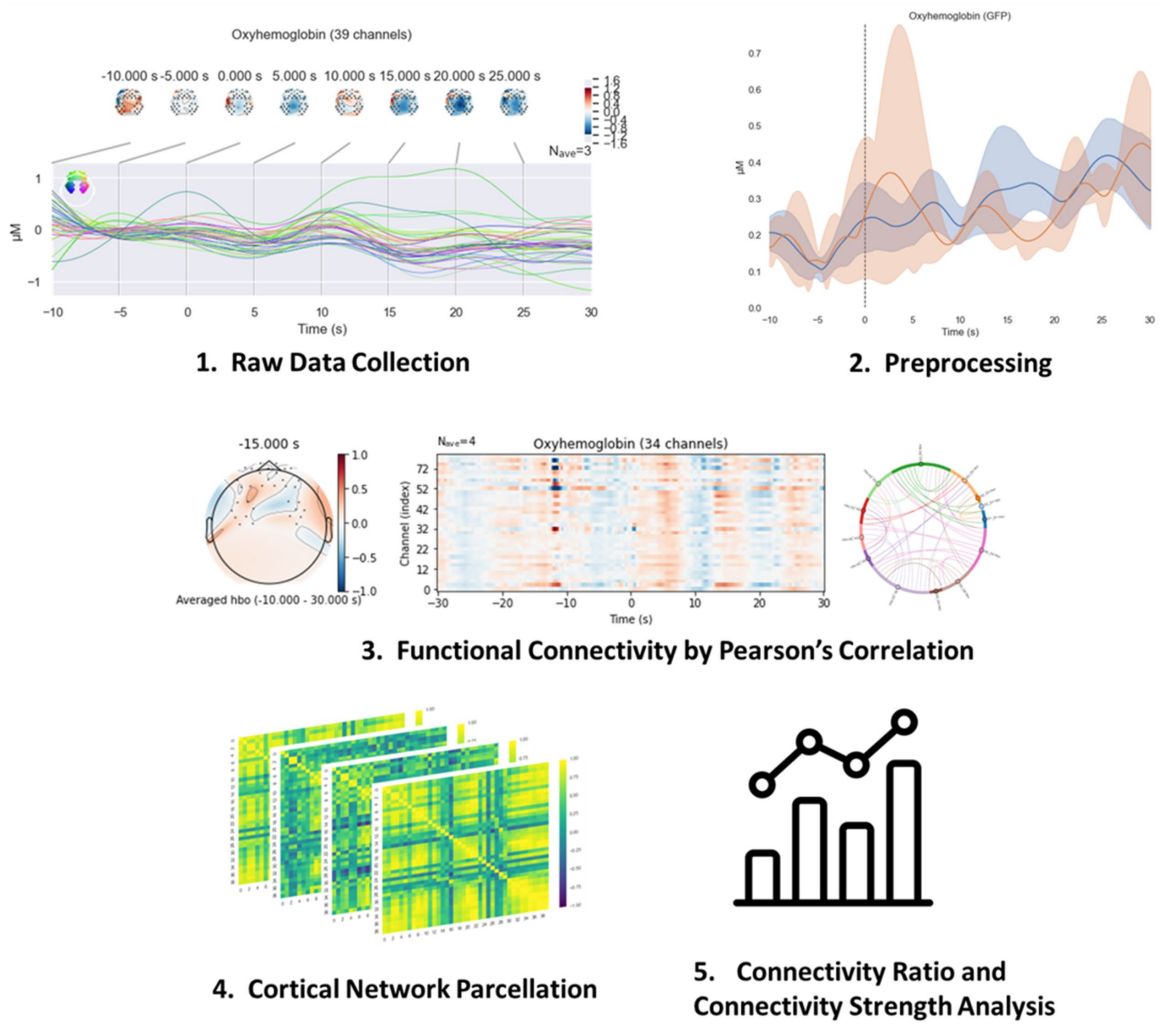
fNIRS neuroconnectivity analytical pipeline.

fNIRS data were preprocessed with MNE (Gramfort et al., 2013). Figure 4 shows the analysis pipeline. The time-series fNIRS data were read into the software and converted into optical density. Next, we measured the connection quality between the optode and the scalp through the scalp coupling index (SCI) (Pollonini et al., 2014). Channels in experiment trials with SCI less than 0.5 were marked as channels with low data quality and removed. The corrected signal was then bandpass filtered for a frequency range of 0.04 Hz–0.15 Hz (Nguyen et al., 2018) to remove physiological noise, with a transition bandwidth of 0.1 Hz and 0.02 Hz at the high and low cut-off frequency. Then, the filtered optical density signal was converted to the oxygenated and deoxygenated hemoglobin concentration according to the Beer–Lambert law with a partial path length factor of 6. Although fNIRS data can be analyzed with multiple measures such as oxy-hemoglobin (HbO), deoxy-hemoglobin (Hb), and total hemoglobin (THb), HbO was selected for further analysis because it is the most sensitive indicator to cerebral blood flow changes in motor-related tasks (Hoshi et al., 2001). We utilized the signal space projection (SSP) to suppress interference and noise in electromagnetic signals. After completing the preprocessing for fNIRS, we performed baseline correction using the resting-state data collected at the beginning of the experiment. In our analysis, we focused on the pick-up events (for continuous 40 s worth of data prior and post picking up of the cubes), given its motor difficulties in the entire teleoperation task presented with delays. The pick-up events were marked in fNIRS data during the experiment. Each continuous trial was sliced into 40-s epochs, starting at 10 s before the events and ending at 30 s after the events.

The Pearson correlation coefficients (*ρ*) were calculated between each pair of fNIRS channels for each epoch. The ρ values for a subject in each condition were the average of all ρ values calculated from the epochs belonging to this subject and this condition. In this experiment, the data generated a 40 × 40 ρ matrices for each epoch. Then, the ρ values were converted to a normal distribution following standard processing pipeline (Zhu et al., 2021) through the Fisher z-transformation (Corey et al., 1998) using Equation 5 below:


(5) $$z=\frac{1}{2}\ln\frac{1+\rho }{1-\rho }$$

To deliver a more comprehensive evaluation, a cortical network parcellation was performed to divide the channels into two sub-networks in the Brodmann area: the prefrontal cortex (PFC, 20 channels) and the motor cortex (M1, 20 channels). Finally, four cortical networks were formed: three within-networks (full connection, PFC, and M1) and one inter-network (PFC-M1). The full connection network is 40 × 40 matrices, and the other networks (PFC, M1, and PFC-M1) are 20 × 20 matrices.

Two features were extracted from the network metrics to perform the statistical analysis: connection ratio (CR) and connection strength (CH) (Nguyen et al., 2018). CR was calculated as the ratio of significant connections over all connections in a network. The significant connection was defined as the pair of channels with an absolute *z*-value greater than a threshold of 0.3 in this experiment. CS was defined as the average strength of connections in a network, calculated by the sum of absolute *z* values divided by the number of connections.

The number of significant connections in a network was calculated by counting all connections belonging to the network that had an absolute *z*-value greater than the threshold. In this study, the threshold range was chosen from 0.2 to 0.7. The connection ratio (CR) was then computed as a ratio of the significant connections on the network total connections. A network’s connection strength (CS) was the average of the absolute *z*-values of all connections.

### 3.7 Statistical analysis

The variables were individually first checked for normality, and those whose distributions differed from normality were transformed to a normal distribution using logarithmic transformation. Normally distributed variables were checked for homogeneous variances. Variables that passed the assumption checks were analyzed using ANOVA on parametric study measures. T-tests with Bonferroni corrections were used for *post hoc* pairwise comparison for each condition pair. Signed rank Wilcoxon tests were performed to examine any significant differences for non-parametric data distributions, including CR and CS. The statistical tests were considered statistically significant when the *p*-value was less than 0.05.

## 4 Results

### 4.1 Performance and perceived latency

**Figure 5:**
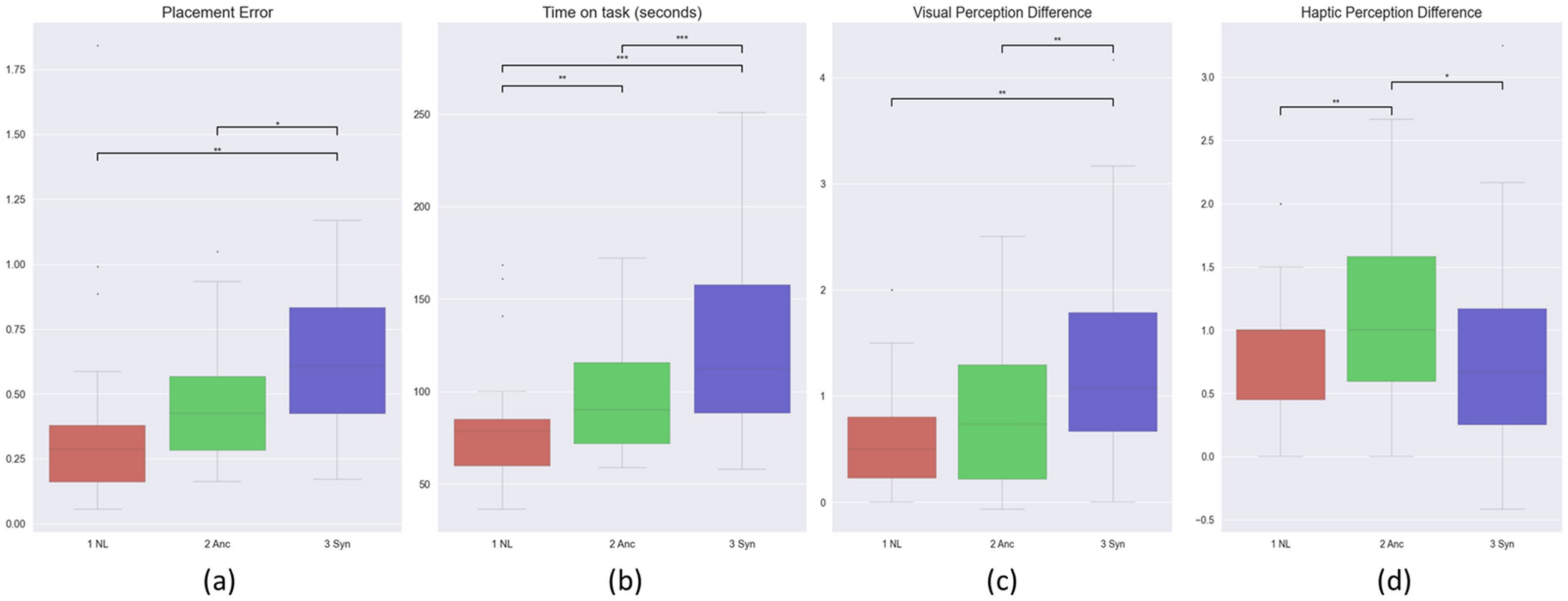
Teleoperation performance measured by **(A)** placement error, **(B)** time on task comparison; Perceived latency accuracy: **(C)** visual perception difference, **(D)** haptic perception difference. *, **, and *** denote a pairwise comparison with 0.05 > *p* > 0.005, 0.005 > *p* > 0.0005, and 0.0005 > *p*.

Figure 5 shows the result of performance and perceived latency. The results indicate significant differences in placement accuracy between the NL and the Syn conditions (*p* = 0.004) and between the Anc and Syn conditions (*p* = 0.032). We found that the Anc condition, i.e., providing real-time haptic cues, significantly improved the hand-picking task regarding placement accuracy. The benefits could be because participants could rely more on haptic feedback when it was available to coordinate the teleoperation actions. The advantage of providing real-time haptic stimulation boosted the performance to a level similar to the NL condition (*p* = 0.168). As for time on task, the results also indicate significant differences between the NL condition and the Syn condition (*p* < 0.001) and between the Anc condition and the Syn condition (*p* = 0.049). While the Anc condition did not perform as well as the NL condition (*p* = 0.009), it still outperformed Syn conditions regarding time on task.

As for the perceived latency, while participants generally overestimated the latency, the results suggest that the Anc method could reduce the subjective feeling of teleoperation latencies (visual delays) by up to 1 s. Here we focused on examining if the proposed sensory manipulation method could reduce perceived visual delay, which is considered the most common and troublesome delay in robot teleoperation. The data shows that the overall average perceived visual delay in teleoperation under the Anc condition was significantly lower than the Syn condition. In addition, 18% of participants reported a perceived visual delay that was minor compared to the actual one under the Anc condition. It implies that coupling real-time haptic feedback with the action during teleoperation can mitigate the subjective feeling of delays. For example, when the actual visual delay was 750 ms, a subject reported 100 ms as the perceived delay. Figures 5C,D compare the haptic, visual perception difference, and the visuomotor gap perception difference. As for the perceived haptic delays, the data shows a slightly different pattern. Subjects seemed to report a lower perceived haptic delay under the Syn condition. This makes sense because the coupled haptic and visual feedback may help to estimate the haptic delay better. As for the visuomotor gap perception, it shows that under the Anc condition, many subjects reported a delay more minor than the actual one. All these results confirmed the perceptual benefits of having real-time haptic feedback.

### 4.2 Neural connectivity

#### 4.2.1 Connectivity ratio

**Figure 6:**
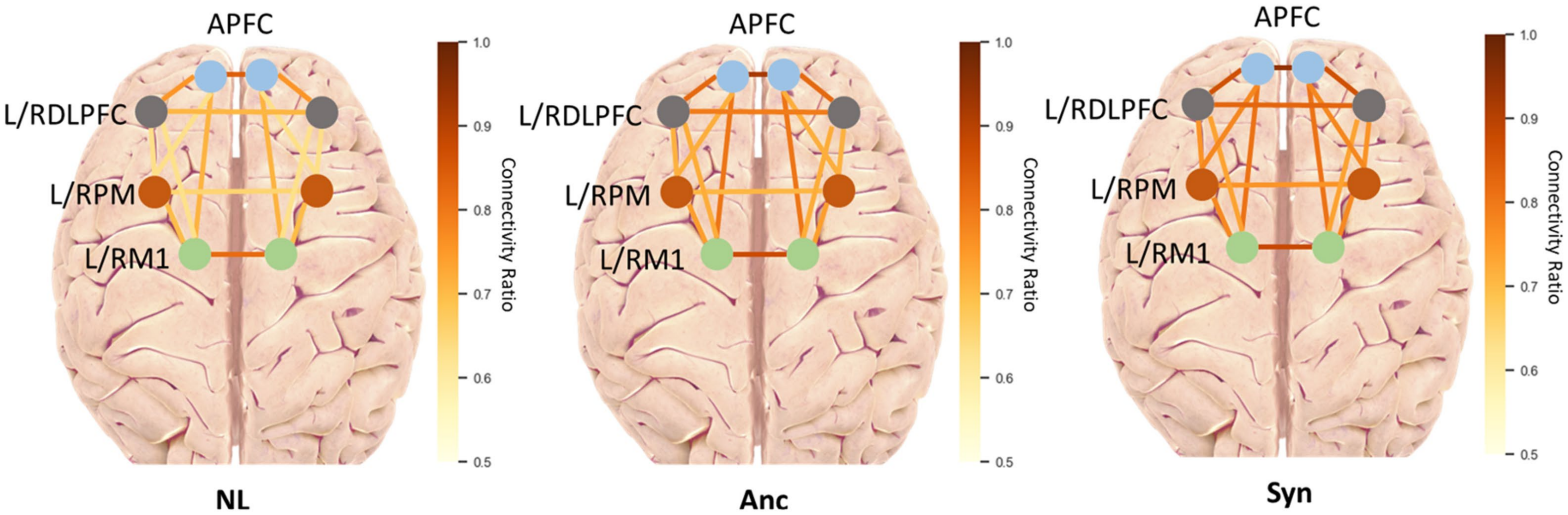
Average fNIRS CR values in all sub-networks.

The CR threshold of 0.3 was chosen because previous studies (Nguyen et al., 2018) showed that 0.3 preserved a reasonable detail of connectivity patterns. Figure 6 visualizes the average CRs across all subnetworks in the three teleoperation conditions. In general, the presence of latency increased the CRs, as demonstrated by the comparison between the NL and Syn conditions. The Syn condition led to the highest CR, and the NL condition led to the lowest CR. The CRs in Anc condition were in between.

**Figure 7:**
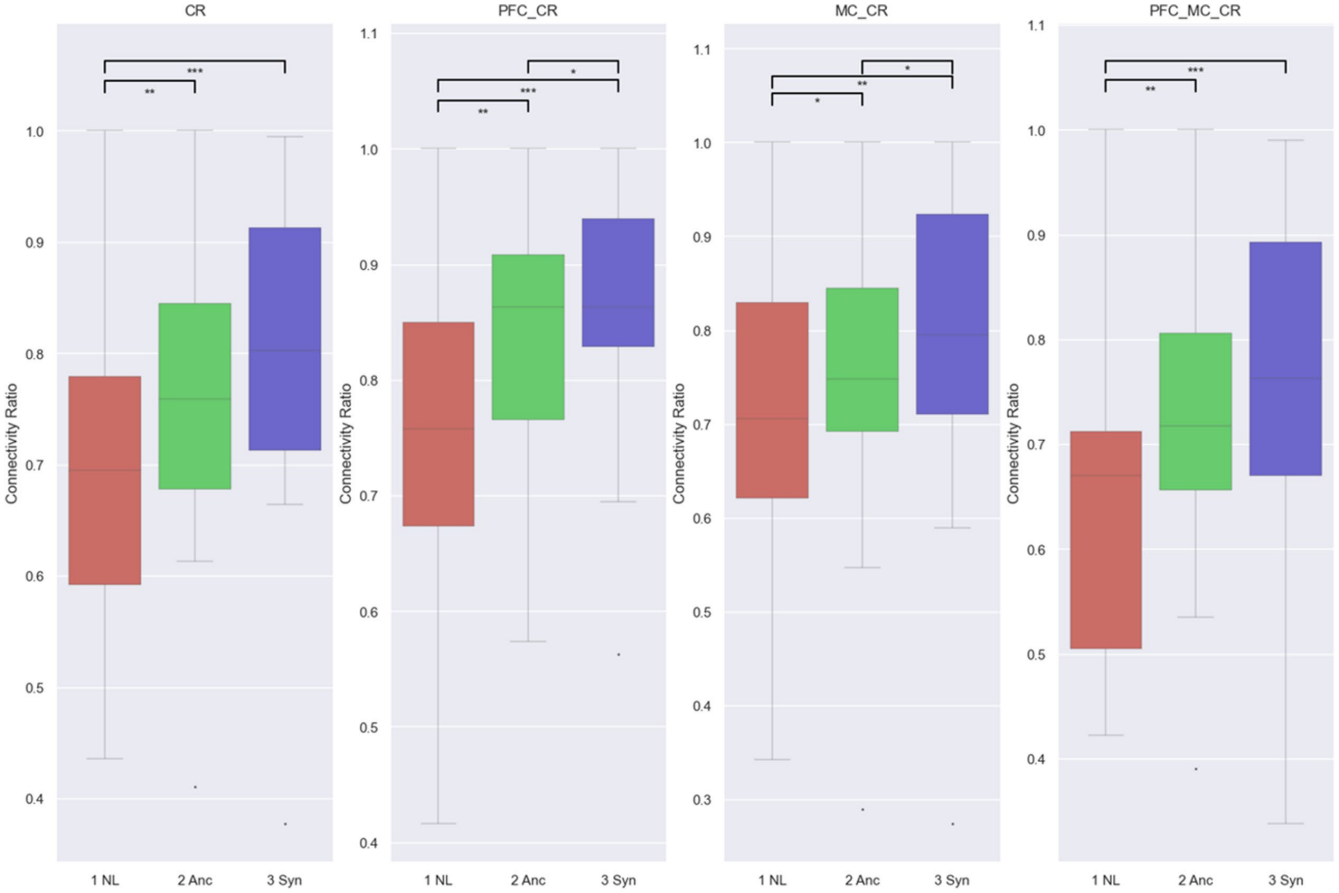
fNIRS CR from HbO, with CR threshold = 0.3. *, **, and *** denote a pairwise comparison with 0.05 > *p* > 0.005, 0.005 > *p* > 0.0005, and 0.0005 > *p*.

To provide further insights, we aggregated sub-networks into PFC and M1 for statistical analysis. Figure 7 displays the connection ratios (CR) derived from HbO for four networks in three conditions. Specifically, the participant’s CR in the optimal teleoperation condition, NL, was significantly different with Anc and Syn in all networks of interest: full connection (*p* = 0.001; *p* < 0.0005 compared with Anc and Syn), PFC (*p* = 0.002; *p* < 0.0005), M1 (*p* = 0.021; *p* = 0.001), and PFC-M1 (*p* = 0.002; *p* < 0.0005). Meanwhile, providing haptic feedback of lower latency led to significantly lower CRs compared with the synchronized haptic and visual latency within the PFC (*p* = 0.028) and M1 (*p* = 0.035) networks. Although the CRs of the full connection network and between PFC and M1 were not significantly different between Anc and Syn conditions, the *p* values were comparatively small (*p* = 0.073 for the full connection network, *p* = 0.238 for PFC-M1 network) and the average CRs were lower in Anc condition.

#### 4.2.2 Connectivity strengths

**Figure 8:**
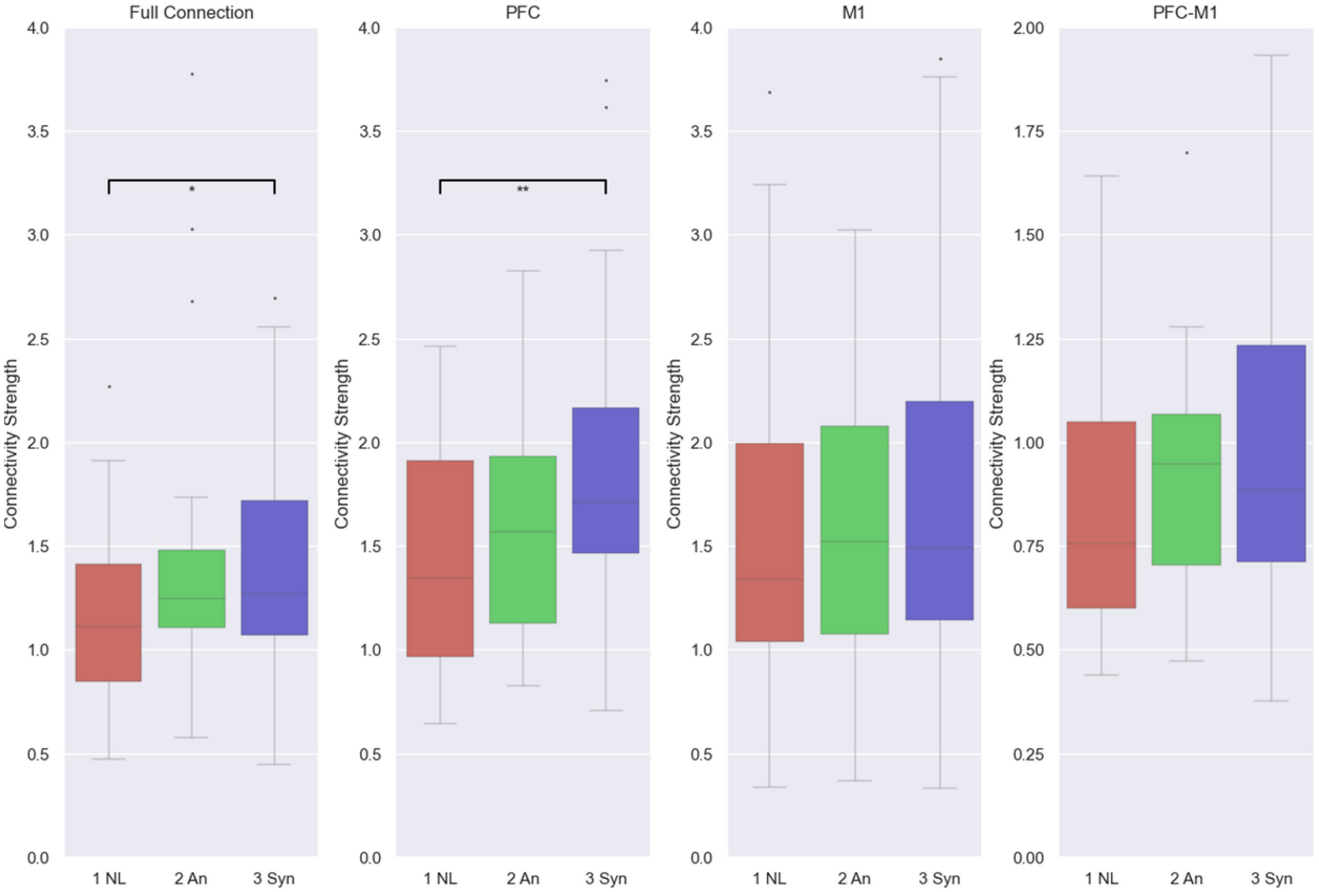
fNIRS CS from HbO. *, **, and *** denote a pairwise comparison with 0.05 > *p* > 0.005, 0.005 > *p* > 0.0005, and 0.0005 > *p*.

Average connectivity strengths calculated from HbO were generally similar, except between the NL and Syn conditions in the full connection network (*p* = 0.0076) and PFC network (*p* = 0.0036). Like the CR results, the average connectivity strengths were the lowest. At the same time, no latency was presented (NL condition) and highest while the same high latency for haptic feedback and visual feedback was shown (Syn condition) (Figure 8).

## 5 Discussion

We designed an experiment to gain neurological evidence on the impact of teleoperation latency on human cognitive activities. The brain functionality networks were investigated under different teleoperation latencies: no latency (NL), real-time haptic feedback and full-delayed visual feedback (Anc), and haptic and visual feedback with synchronized latency (Syn). ANOVA and *post hoc* tests showed that the brain functional connectivity was strong in the Syn condition and weaker in the NL condition among all brain regions of interest (overall connection, within PFC, within M1, and between PFC and M1). This result provides solid neurological evidence that latency can significantly impact the neurological path and coordination among brain regions.

In particular, a statistically higher CR was expressed in all networks, and a significantly stronger CS was shown in the full connection and PFC networks in the Syn state. The increased functional connectivity within the PFC indicates a potential increase in cognitive load and anxiety level (Zhang et al., 2022), which echoes previous operation latency studies (Yang and Dorneich, 2017). The high functional connectivity between PFC and M1 implies the more intensive involvement of neuropaths that coordinate the perception and motor control functions, which can be interpreted as the increased difficulty of motor control and decision-making in this motor-intensive teleoperation task (Koch et al., 2006). Meanwhile, functional connectivity within M1 increased as the latency was introduced. This behavior implies more intensive involvement of motion selection and preparation (Koch et al., 2006) when latency is introduced. Taking the results together, this experiment showed neurological evidence that latency in teleoperation could induce cognitive burden, anxiety, and more challenges in high-level and motor-control decision-making. These mental activities, unrevealed by fNIRS data, coincide with the performance results, which show that latency reduces teleoperation accuracy and speed.

Moreover, we evaluated the cognitive impact of a latency mitigation method that provides real-time haptic feedback to simulate a pseudo-real-time haptic perception for a more coordinated action-haptic perception loop (Du et al., 2023). This latency mitigation method aims to manipulate the operator’s subjective perception of latency and achieve better performance and subjective feelings. The Anc condition in this experiment adopted such a mitigation principle. In general, results showed that the Anc condition reduced the CR and CS compared to Syn, attributing to the reduced average values and distribution statistics. In other words, Anc condition pushed the neurological patterns to a state closer to the no-latency teleoperation. Together with the performance improvement, the results provide neurological evidence that the mitigation methods can positively influence cognitive activities in the teleoperation task.

Interestingly, when latency is presented in teleoperation, CRs within PFC and within M1 were significantly reduced by the pseudo-real-time haptic feedback (Anc condition) but not between PFC and M1. These functional connectivity changes implied that neuropathy connecting different areas within PFC and within M1 cortexes were less activated, while the communications in between remained relatively intensive. A potential interpretation is that while maintaining a similar level of information exchange between decision and action, the Anc condition led to a paradigm shift in the neural process, in which operators relied more on the sensorimotor process, thus reducing the cognitive activity and motion decision load (Bardouille and Boe, 2012). This interpretation echoes the observation that participants reported less perceived visual delay in the Anc condition.

Limitations of this study need to be addressed to better understand the scope of generalizability of the results and to give direction to similar investigations in the future. First, this study presented evidence on the neural mechanism of latency-induced performance in teleoperation tasks. While these findings are essential, further studies are needed to investigate whether similar neural change patterns are valid for more complex teleoperation tasks and latency-mitigation methods. These mechanistic investigations are critical for designing future HMIs that understand and mitigate latency issues. Second, the experiment in this study was conducted as a single 2-h session for each participant, which did not capture the learning effect and residual effect. Future studies are needed to perform tests over a period of time to examine the potential adaptation effect to teleoperation under latency.

To address these limitations, future research should investigate the neural impact of latency in more complex and varied teleoperation scenarios, such as remote surgery, underwater exploration, and space robotics, to determine if the observed neural patterns generalize across different contexts. Additionally, conducting longitudinal studies with multiple sessions spread over weeks or months will help capture the learning effects, adaptation, and residual impacts of latency on performance and cognitive load. In the further future, we plan to apply these findings and technics to real-world teleoperation tasks to validate the practical applicability of using enhanced sensorimotor processes to mitigate latency. We will provide insights into how professional operators, such as surgeons and engineers, are affected by latency in terms of performance, workload, and potential adaptation. By addressing these areas, future research can provide a more comprehensive understanding of the cognitive and neural impacts of latency in teleoperation, ultimately leading to the design of more effective and ergonomic teleoperation systems to mitigate negative impacts induced by latency. For example, future work could inspire low cognitive-burden remote surgery for broader access to expert surgeons as well as contribute to the long-distance space robot teleoperation for scientific exploration. Overall, by examining the neural processes and cognition-friendly designs related to teleoperation latency, this research can contribute to advancements in critical sectors, enhancing both individual well-being and societal progress.

## 6 Conclusion

This study reported how the operators’ neural processes were impacted by the teleoperation latency and a latency mitigation method through functional connectivity analysis with fNIRS data. Indicated by increased connectivity ratio and connectivity strengths, the results showed that the presence of latency in teleoperation increased functional connectivity within and between prefrontal and motor cortexes, representing higher levels of cognitive load, anxiety, and challenges in motion planning and control. While maintaining the same visual latency, providing real-time haptic feedback reduced the average functional connectivity in all cortical networks and showed a significantly different connectivity ratio within prefrontal and motor cortical networks. This implies that this latency-mitigation method impacts neural paths, especially those related to the cognitive, decision-making, and sensorimotor processes. The performance results (placement error and time on task) showed the worst performance in the all-delayed condition, which echoes the neural activity patterns. The findings from this study show that neurological evidence that latency in teleoperation is detrimental to operators mainly due to the increased level of cognitive barrier, and creating an optional sensorimotor process could potentially mitigate such an effect.

## Data Availability

The datasets presented in this study can be found in online repositories. The names of the repository/repositories and accession number(s) can be found at: https://www.dropbox.com/scl/fo/4qcvc4v3nxhc9in0oi6bo/h?rlkey=15eds1e7on4vjkstannruok5r&dl=0.
